# All-You-Can-Eat: Influence of Proximity to Maize Gardens on the Wild Diet and the Forest Activities of the Sebitoli Chimpanzee Community in Kibale National Park

**DOI:** 10.3390/ani12070806

**Published:** 2022-03-22

**Authors:** Chloé Couturier, Sarah Bortolamiol, Sylvia Ortmann, John-Paul Okimat, Edward Asalu, Sabrina Krief

**Affiliations:** 1 Eco-Anthropologie (EA), Musée de l’Homme, Muséum National d’Histoire Naturelle, CNRS, Université de Paris, 17 Place du Trocadéro, 75116 Paris, France; sabrina.krief@mnhn.fr; 2 Great Ape Conservation Project (GACP), Sebitoli Research Station, Kibale National Park, Fort Portal, Uganda; sarah.bortolamiol@cnrs.fr (S.B.); paulamugiet@gmail.com (J.-P.O.); 3 Fondation pour la Nature et l’Homme, 6 rue de l’Est, 92100 Boulogne-Billancourt, France; 4 RG Evolutionary Ecology, Leibniz-Institute for Zoo and Wildlife Research, D-10315 Berlin, Germany; ortmann@izw-berlin.de; 5 Uganda Wildlife Authority, Plot, 7 Kira Rd, Kampala, Uganda; easalu@gmail.com

**Keywords:** activity budget, anthropogenic habitat, crop feeding, energy balance, *Pan troglodytes schweinfurthii*, Uganda

## Abstract

**Simple Summary:**

Understanding the resilience of primate populations to the threat of agricultural expansion is critical for effective conservation. Based on individual monitoring from morning to evening of wild chimpanzees in and around a protected area, we showed that the availability of maize at the forest edge had little effect on their activity budget by less resting and no impact on their wild diet and energy expenditure. In this area, large, caloric wild fruits are available year-round, and we observed no behavioral or dietary changes regarding wild resource availability either. Thus, the chimpanzees consume maize opportunistically as a bonus treat in their diet, and the presence of this nutritious resource does not seem to affect their role in seed dispersal and forest regeneration.

**Abstract:**

Frugivorous primates have developed several strategies to deal with wild fruit scarcity, such as modifying their activity budget or enlarging their diet. Agricultural expansion threatens primate habitats and populations (e.g., disease transmission, agrochemical exposure), but it also increases crop feeding opportunities. We aimed at understanding whether maize presence close to the natural habitat of chimpanzees, a threatened species, would lead to significant behavioral modifications. We monitored 20 chimpanzees over 37 months in Kibale National Park, Uganda, with maize gardens at the forest edge. Based on focal nest-to-nest data, we analyzed their diet, activity budget, and energy balance depending on wild fruit and maize availability. We found that the Sebitoli area is a highly nutritive habitat for chimpanzees, with large and caloric wild fruits available all year long. The chimpanzees opportunistically consume maize and exploit it by resting less during maize season. However, no significant variation was found in daily paths and energy expenditures according to maize availability. No behavioral or energy modification was observed regarding wild resources either. Despite the availability of nutritious domestic resources, chimpanzees still exploit wild fruits and do not limit their movements. Thus, their contribution to seed dispersal and forest regeneration in this area is not affected.

## 1. Introduction

Tropical forests are complex habitats with unpredictable fruit availability and large intraspecies and intersite variations. The distribution of trees of the same species can fluctuate in space with clumpy, uniform, or random patterns [1,2], and fruiting patterns can be synchronous or asynchronous [3,4]. Thus, developing effective foraging strategies to survive may affect the behavior of frugivorous species, such as some non-human primates [5,6]. Consequently, their seed-dispersal role may be modified, which may affect the regeneration of already threatened forests and, in the medium term, the quality of the primate diet in terms of diversity and abundance. [7,8].

During periods of low fruit availability, primates may reduce group size by decreasing food competition (*Pan troglodytes* spp. [9,10,11,12]; *Pan paniscus* [9]). They also expand their diet range by consuming fallback foods, i.e., low-quality items that are eaten in large quantity when preferred foods are not available [13]. For example, fibrous items allow frugivorous primates to maintain stable carbohydrate levels, for example, terrestrial herbaceous vegetation (THV) for chimpanzees (*Pan troglodytes* spp. [9,12]), bark for orangutans (*Pongo* sp. [14]), or multiple vegetal parts for neotropical primates (*Ateles belzebuth, Lagothrix lagotricha, Cebus apella*, and *Alouatta seniculus* [15]). However, due to their lower nutrient quality, fallback foods must be consumed in large quantities to supplement fruit intake, which alters the activity budget by increasing daily feeding and travel time at the expense of resting time (*Pan troglodytes verus* [16]; *Alouatta palliata mexicana* [17]). In addition to these seasonal variations, there are interindividual differences in activity budget and energy balance based on sex, age, weight, reproductive status, or dominance rank [18,19,20].

Today, agricultural expansion is one of the major threats to tropical forests, the main habitat of great apes [21,22], which often live outside protected areas and may use forest–farm mosaics and human-dominated landscapes for foraging [23,24,25,26]. Studying energy balance may help us to understand how modified and threatened habitats may affect great apes, especially since these species are all classified as endangered or critically endangered [27] and have slow life history traits. Great apes have their first offspring between 10 and 16 years old [28] and long interbirth intervals of 3 to 8 years [29]. These features make it difficult to rapidly assess population sustainability through censuses and demographic surveys, especially in fast-changing environments.

Crops represent easily digestible and nutritive foods rich in carbohydrates [30,31] sparsely dispersed in space and with high seasonality. Besides physiological consequences related to the potential increase in energy intake and food diversity [32], this direct proximity with humans also affects primate foraging strategies and behavior as they cope with stress [33,34] and avoid predation and risks [35,36]. In periods of crop maturity, apes living within farm–forest matrices increase their travel time at the cost of resting (*Pan troglodytes* verus [37]; *Pongo abelii* [38]), whereas other primates reduce travel time and increase rest time (*Chlorocebus aethiops pygerthrus* [39,40]; *Macaca silenus* [41]; *Papio anubis* [42]). Proximity to crops can also encourage chimpanzees to develop nocturnal activities to avoid field guarding (*Pan troglodytes schweinfurthii* [43]).

Chimpanzees, an endangered species [27,44], are mainly frugivorous with a flexible diet that highlights fast adaptation through cognitive skills [45]. Due to the expansion of agriculture, their home range may be close to gardens, providing opportunities to exploit these nutritious resources (see [46,47] for reviews), even if this behavior represents a high-risk activity [36,48,49].

Although information regarding energy balance is useful to better understand the threats affecting endangered species, accurate assessment of the energy expended and assimilated is complicated. First, it requires an ethical, non-invasive approach. Capturing and darting individuals represents significant health risks (injury due to fall from a tree, disease transmission, etc.) and may bias physiologic markers due to stress and excessive movements during the capture process. In addition, such invasive methods interfere with the habituation process, monitoring, and well-being of the community [50,51,52]. Second, methods to estimate energy balance are not adapted to remote study sites and inaccessible species (vegetation, topography, etc.) or unhabituated individuals. Despite such difficulties, Pontzer and Wrangham [53] estimated the energy cost of chimpanzee traveling and climbing, and N’Guessan et al. [54] highlighted seasonal variations in chimpanzee energy balance by combining direct observations with equations adapted from human studies. However, the literature on wild apes remains relatively sparse.

In this study, we aimed to improve our understanding of how the activity budget and energy balance of wild chimpanzees vary with maize presence and abundance of wild fruits. We hypothesized that chimpanzees would be opportunistic and consume maize because of its spatial and temporal availability (clustered gardens at the boundaries of the protected area with synchronized maize maturity), regardless of fruit availability in the forest. In this case, when maize is available, we expected a lower proportion of wild fruits in the chimpanzees’ diet and thus lower energy gains from forest fruits—a pattern similar to that observed during periods of wild fruit scarcity. By eating more nutritious crops, chimpanzees will also reduce their foraging effort, meaning more rest, less travel, and thus lower energy expenditures—a pattern similar to that observed during periods of high wild fruit availability. Finally, regardless of maize gains and given how chimpanzees exploit wild resources, we expected similar energy balances between maize and non-maize seasons as a result of reduced wild intakes and expenditures in the former case but increasing them in the latter. An alternative hypothesis could be that chimpanzees only use maize as a fallback food when wild fruit availability in the forest is low.

To test this hypothesis, we studied a chimpanzee community (*Pan troglodytes schweinfurthii*) living at the northern extremity of Kibale National Park, Sebitoli (Uganda) in an area known as Sebitoli. There, 82% of the chimpanzees’ home range is surrounded by agricultural activities, including subsistence gardens at the direct forest border [55]. Maize cob is the main and almost only crop item consumed by the chimpanzee community, and farmers usually cultivate it with high seasonality twice a year [56].

## 2. Materials and Methods

### 2.1. Study Site

Kibale National Park (KNP) is a protected area of 795 km^2^ located in southwestern Uganda (0° 13′–0° 41′ N; 30° 19′–30° 32′ E) and composed of mature forest, grass lands, swamps, and regenerating forest mosaic. KNP is well-known for its rich diversity of plants and mammals, including more than 1000 individual threatened eastern chimpanzees (*Pan troglodytes schweinfurthii*) living in different communities [57].

A high human population density is present at the edge of the forest (up to 335 inhabitants/km^2^ [58]), as the park is surrounded by tea estates, eucalyptus plantations, and small farms with cash and subsistence crops [59,60]. Maize (*Zea mays*) is usually cultivated and harvested by farmers twice a year following the rotation of two wet (March–May and September–November) and two dry seasons (December–February and May–August) [56,61].

**Figure 1:**
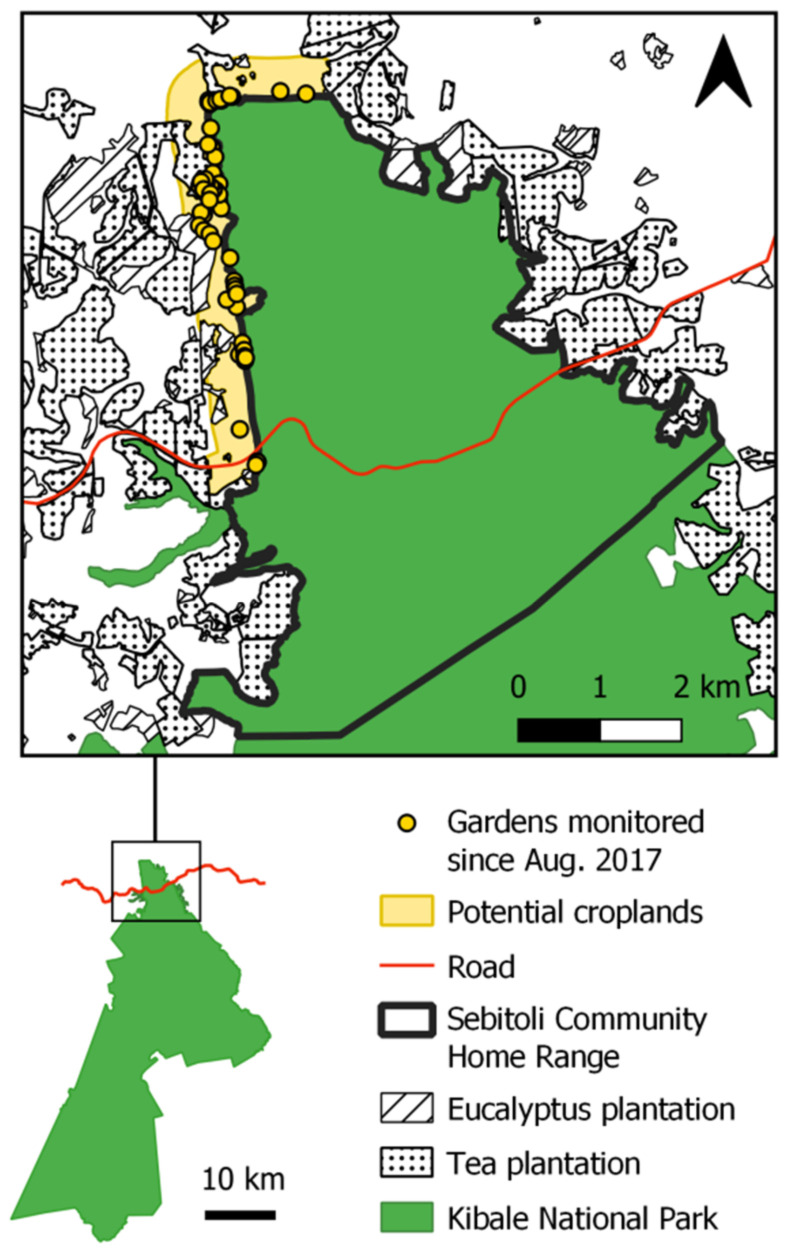
Location of maize gardens between August 2017 and January 2019 (*n* = 72) at the edge of the home range of Sebitoli chimpanzee community, Kibale National Park, Uganda (sources: SCP, S. Bortolamiol, C. Couturier).

Located in the extreme north of KNP, Sebitoli area, defined as the home range of the Sebitoli chimpanzee community, is a forest patch covering 25 km^2^, bisected by a high-traffic national road and contiguous with agriculture on its western, eastern, and northern boundaries [55,62,63]. The Sebitoli area was commercially logged from 1950 to the 1970s, leading to damage of about 50% of the trees; today, degraded or regenerating forests represent 70% of this area, and only 14% represents old-growth forest [60]. All crop fields are outside the national park, along the northwestern forest border (Figure 1).

### 2.2. Sebitoli Chimpanzee Community

The Sebitoli Chimpanzee Project (SCP) started chimpanzee habituation in the Sebitoli area in 2008, and 12 years later, this research team is composed of 25 Ugandan field assistants, eight of whom follow chimpanzees daily, along with researchers and students. The chimpanzee community size is estimated to be 100 individuals, 60 of which are regularly monitored on a 25 km^2^ territory across the national road [63]. Each chimpanzee is identified with a name and a two-letter code, and its age and birth date are estimated or recorded when possible. Age classes (adult, subadult) were defined according to Pontzer and Wrangham [64]. The sex ratio of known individuals is 1 male for 1.15 females, and more than 25% of the individuals have disabilities [65]. Having previously assessed the birth date of the infants by direct observations (date of absence of the females and date of return with a newborn), we distinguished lactating and gestating mothers (MO) from non-maternal females (AF), i.e., non-pregnant females or without a dependent infant, by estimating the mean duration of chimpanzee gestation as 32 weeks [66,67,68] and to 5 years for the lactation period [69].

### 2.3. Wild Fruit Availability

Between January 2016 and January 2019, temporal wild fruit availability was evaluated by monthly phenology surveys on 10 transects, each 500 m long, distributed through the Sebitoli chimpanzee community home range [70]. As many as 445 trees from 46 species known to be eaten by chimpanzees according to long-term SCP data were monitored. For each tree, we attributed a score from 0 (no item) to 4 (maximum) to describe the abundance of fruits, leaves, and flowers. We calculated a monthly food availability index (FAI) for wild fruits only adapted from Hockings et al. [30]:

(1) $$\mathrm{FAI}=\frac{\sum G_{i}\times {\;F}_{i}\;}{\sum G_{i}\times 4\;}\times 100$$

where G_i_ is the basal area of the tree, i, and F_i_ is its abundance score for an item. Some favorite species for chimpanzees were absent from plots or presented a clumpy distribution, such as *Mimusops bagshawei* [55]. We preferred this FAI index, already used in the study area by Bortolamiol et al. [60], to those including tree density from plots [70,71]. We included ripe fruits from all species and unripe fruits from *Ficus sur, Ficus exasperata, Ficus natalensis*, and *Mimusops bagshawei*, which are known to be consumed when ripe and unripe by the Sebitoli community according to SCP long-term data. Due to missing data, the FAI was not calculated for 3 out of 37 months (April 2016; July 2017; September 2017). We distinguished high fruit availability months (HFA), i.e., months with FAI values greater than or equal to the mean value of the sum of ripe and unripe fruits during the study period, from low fruit availability months (LFA), i.e., months with FAI value less than the mean value.

### 2.4. Maize Availability

Chimpanzees consume both ripe and unripe maize cobs, as well as maize stems [43]. We defined the monthly presence (0/1 score) of maize edible by chimpanzees at the north-western border by computing direct observations, informal interviews with farmers, camera trap data over 2016–2019, and, since August 2017, a census of 72 maize gardens (mean size = 1.1 ha) by three SCP field assistants. On average, maize was considered edible from between 10 and 12 weeks after sowing to harvest (up to 27 weeks).

### 2.5. Monitoring of Individuals

Between January 2016 and January 2019, each day, one chimpanzee of the community was selected and monitored according to Altmann’s focal animal sampling [72] on a nest-to-nest basis, described as FNN below, usually from 6:00 am to 6:30–7:00 pm. The focal individual was chosen among the better-habituated adults and subadults as soon as individuals present in the party were identified. When possible, we avoided choosing the individuals already selected during the last 4 days of monitoring. If an individual fitting those criteria was observed before 12:00 am, then the observer could start an FNN. The observer recorded each activity and the time spent with a focus on alimentation and movements in trees (see energy sections below) but also detailed the substrate used and its firmness, as well as the health condition of the focal individual.

The focal individual was considered lost after 45 min in the absence of any present evidence of the party followed (paths or vocalizations) [53]. During feeding sessions of the focal individual, the ingestion frequency of a given item, i.e., the number of fruits/leaves or the length of the stem of the species eaten in one minute, was counted by the observer every 10 min from the beginning to the end of the session to cover all periods of satiety [53]. Behavior monitoring was associated with spatial monitoring. The position was automatically recorded every 30 s by a GPS Garmin^®^ (Nanterre, France) 64CS (hereafter called GPS tracks) held by the observer, who, as far as possible, followed the exact chimpanzee paths.

### 2.6. Energy Expenditures

We decided to approximate the seasonal energy balance with direct field observations of the activity budget. Because of the lack of literature using this methodology on chimpanzees or other great apes, we relied primarily on the approaches of N’Guessan et al. [54] and Pontzer and Wrangham [53] to determine expenditures and intakes and for comparison purposes.

The daily energy balance corresponds to the difference between gains provided by the food resources ingested and expenditures lost by the organism. The total daily energy expenditure (*TDEE*) is composed of the basal metabolic rate (*BMR*), i.e., the energy required to maintain vital functions of the organism, such as breathing or digestion [73], and the amount of energy required to realize physical activities (*E_i_*):

(2) $$TDEE=BMR\times \Sigma \;E_{i}\;\left(\mathrm{kcal}\right)\;$$

We applied a 1.25 factor for gestating females and a 1.5 factor for lactating females to the *TDEE* [18].

The daily *BMR* (in kcal) was calculated by using Kleiber’s equation [74] (used in [53,54]) based on the individual body mass, *M_b_*:

(3) $$BMR=70\times M_{b}^{\;0.75\;}\left(\mathrm{kcal}\cdot {\mathrm{day}}^{-1}\right)$$

We used body mass values for *P.t. schweinfurthii* estimated by Smith and Junger [75] (used in [53]): 43 kg for adult males and 36.9 kg for adult females. We added an additional weight of 5 kg for mothers with a dependent infant [54]. We divided the 150 detailed behaviors observed during FNN into six categories: feeding (F), resting (R), moving in trees (M), and traveling (T). For social activities (SA), we distinguished high social activities (HSA) from low social activities (LSA) [76,77]. An ethogram is available in Table 1. If two activities were simultaneously realized and recorded, we selected the one with the highest value in terms of energy (gain or expenditure).

#### 2.6.1. Daily Traveled Distance and Traveled Energy

The GPS tracks associated with the FNNs were processed with ArcGIS^®^ 10.2.2 using “Elevation Profile” and “ET GeoWizards” add-ins to extract the exact daily length traveled (DLT), which includes slopes.

To calculate the energy expenditures required for traveling, we used Taylor’s equations [78] based on the theoretical volume of oxygen consumed for walking with a speed, *v_T_*:

(4) $$VO_{2}\left(E_{T}\right)=\left(0.523\times M_{b}^{-0.298}\times v_{T}\right)\;+\;\left(0.345\times M_{b}^{-0.157}\right)\;\;\;\;\;\;\;\left(\mathrm{mL}\cdot {\mathrm{kg}}^{-1}\cdot s^{-1}\right)$$

The traveling speed on the ground, *v_T_*, was estimated by Hunt [79], with 0.88 m·s^−1^ for adult males, 0.78 m·s^−1^ for adult females, and 0.75 m·s^−1^ for adult females with a dependent infant. Energy in kcal was calculated by assuming that 1 L of O_2_ requires 4.8 kcal to be assimilated [78]:

(5) $$E_{T}=VO_{2}\left(E_{t}\right)\;\times \;10^{-3}\;\times \;4.8\;\times \;\frac{M_{b}{}_{\;}}{v_{T}}\;\left(\mathrm{kcal}\cdot m^{-1}\right)$$

#### 2.6.2. Moving in Trees

We distinguished the energy, *E_A_*, required for vertical movements (i.e., ascending or descending, hanging on vertical branches or on a trunk) from the energy, *E_M_*, required for horizontal movements (i.e., hanging or walking horizontally and less than 22.5° inclined branches). Distances were estimated by the observer, referring to the average forelimb length of adult chimpanzees above 50–60 cm [80] and tree height. Field assistants regularly tested each other to assess their height-estimation accuracy.

Mermier et al. [81] suggested that the volume of oxygen consumed by humans for ascension is equivalent to a walking speed of 1.9 m·s^−1^. This assessment was tested on wild chimpanzees by Pontzer and Wrangham [53] and used by N’Guessan et al. [54] in the following equations:

(6) $$VO_{2}\left(E_{A}\right)=\left(0.523\;\times \;M_{b}{}_{\;}^{-0.298}\;\times \;1.9\right)\;+\;\left(0.345\;\times \;M_{b}{}_{\;}^{-0.157}\right)\;\;\;\;\;\;\;\;\;\;\left(\mathrm{mL}\cdot {\mathrm{kg}}^{-1}\cdot s^{-1}\right)$$

(7) $$E_{A}=VO_{2}\left(E_{A}\right)\;\times \;10^{-3}\;\times \;4.8\;\times \;\frac{M_{b}{}_{\;}}{v_{A}}\;\;\;\;\;\;\;\;\;\left(\mathrm{kcal}\cdot m^{-1}\right)$$

where *v_A_* is the ascending speed in trees for chimpanzees, which was estimated by Pontzer and Wrangham [53] as 0.5 m·s^−1^.

To calculate the energy required for horizontal moves in trees, we applied the same equations as for traveling, considering the same speed.

#### 2.6.3. Other Activities

We used the equation below with energetics coefficients (*D*) relative to each activity, *i*: 1.25 for resting, 1.38 for feeding and low social activities, and 2.35 for high social activities [76,77].

(8) $$E_{i}=D_{i}\;\times \;BMR\;\;\;\;\;\left(\mathrm{kcal}\cdot s^{-1}\right)$$

### 2.7. Uphill Grade Approximation

Since the Sebitoli community ranges in mid-altitude mountains with deep valleys, we sought to predict energy expenditures required for elevation changes and slopes during travel (*E_w_*). Thus, we selected Bobbert’s equation [82], which is a logarithmic relationship between *E_w_* (in cal·kg^−1^·min^−1^); the travel speed *v_T_* (in m·min^−1^); and α, the mean positive slope in degrees (°):

(9) $$\log\left(E_{w}\right)=1.427\;+\;\left(0.004591\;\times \;v_{T}\right)\;+\;\;\left(0.024487\;\times \;\alpha \right)+\left(0.0002658\;\times \;v_{T}\;\times \;\alpha \right)$$

However, because of a different relationship between expenditures, speed, and body mass than in Taylor’s equation [78] (4), these results were used only for approximation and comparison and were not included in the interseason analyses.

### 2.8. Energy Gains

We focused our analysis on 13 fruits known to be consumed by the Sebitoli chimpanzee community with nutritional data available for the study site (Table 2). Fruit collection and the drying process were realized in 2015 by S. Bortolamiol following Rothman et al. [83], and dried samples were analyzed by S. Ortmann (see Supplementary Materials). We calculated the caloric gains ingested per FNN as below:

(10) $$Gains=\Sigma {\left(ingestion\;frequency\right)}_{j}\;\times \;{\left(time\;spent\right)}_{j}\;\times \;{\left(\frac{DM}{fruit}\right)}_{j}\;\;\times \;{\left(\frac{kcal}{gDM}\right)}_{j}\;\;\;\;\;\;\;\;\;\;\left(\mathrm{kcal}\right)$$

where *j* is the fruit species, and DM is the dry matter.

### 2.9. Statistical Analysis

Following the central limit theorem [89,90], we assumed that large samples approximate a normal distribution; otherwise, we evaluated the normality of small samples with the Shapiro–Wilk normality test. We tested the hypothesis of opportunistic maize consumption with a two-sample Student’s t-test to compare wild food availability between maize and non-maize seasons.

Activity budget was considered as a percentage of observation time, focusing on the three main diurnal activities: feed, rest, and travel. Frugivory was considered the percentage of wild fruit ingestion time over total feeding time. Daily length traveled (DLT), total energy expenditures (TDEE), and energy balance were analyzed on an hourly basis. We only selected FNNs with a total duration greater than 6 h, corresponding to a half day of monitoring, to analyze frugivory, activity budget, DLT, and TDEE. Due to the lack of nutrition data, we only selected FNNs of 6 h or more when the 13 food items studied covered at least 80% of feeding time to analyze the ingestion rate, i.e., kcal per minute of feeding, and energy balance.

For each response variable, we built a linear mixed-effect (LMM) model including maize availability (maize, non-maize) and wild fruit availability (HFA, LFA) as main effects and their interaction and individuals as a random effects. All assumptions were validated, except residuals normality for DLT, TDEE, ingestion rate, and energy balance, even after basic transformations of our data (log and Box-Cox). By plotting the model residuals per individual, we found extreme intraindividual variations. We therefore tested the significance of the random effect with the *anova()* function between the mixed model and the null model (without random effect). We also built several models including sex and/or age class as covariates and compared their Akaike information criterion (AIC). The random effect appeared to be not significant, and the most appropriate model was the null model. 

As the three activities (feed, rest, and travel) can be correlated and in order to limit type I errors due to multiple tests, we decided to conduct multiple analyses of variance (two-way MANOVA) to assess variations between seasons [91]. Homoscedasticity was tested with Levene’s test, covariance homogeneity with a Box’s M test, and multicollinearity by calculating Pearson’s correlation coefficients for pairwise comparisons. We carried out an analysis of variance (two-way ANOVA) as a post hoc test to assess the effect of seasons on each activity. Then, we ran a two-way ANOVA to analyze DLT, TDEE, ingestion rate, and energy balance between seasons. We used the *t2way()* function and *mcp2atm()* post hoc test from the WRS2 package for a robust ANOVA based on trimmed means [92,93,94]. We used the chi-square test to analyze frugivory among seasons.

Despite our efforts to alternate focal individuals, the FNN sex ratio was rather unbalanced in favor of adult males; thus, we decided to pool male and female data and did not make interindividual comparisons. To understand how individuals contributed to the studied variables, we carried out non-parametric Kruskal–Wallis tests, followed by the Dunn post hoc test with Bonferroni correction between sex–age categories.

A significance level of α = 0.05 was applied, except for the Box’s M test (α = 0.001), and all tests were conducted on R software v.4.0.3 (Vienna, Austria) [95].

### 2.10. Ethical Note

Chimpanzees were observed at a distance of 8 m or more without using invasive methods and without any interaction with the researchers or field assistants. We adhered to the research protocol defined by the guidelines of the Uganda Wildlife Authority and approved by the National Museum of Natural History, Paris, France (Memorandum of Understanding MNHN/UWA/Makerere University SJ 445-12).

## 3. Results

### 3.1. Maize and Wild Fruit Availability

**Figure 2:**
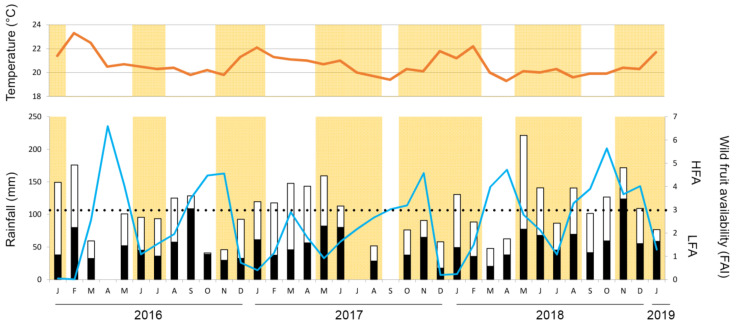
Maize calendar, wild fruit availability, rainfall, and mean temperature between January 2016 and January 2019. The red line represents the mean monthly temperature, and the blue line is the total monthly rainfall (mm). Months with a yellow background correspond to months with edible maize at the PNK edges. Black and white bars represent the monthly wild ripe and unripe fruit availability (FAI), respectively. FAI data were missing for April 2016, as well as July and September 2017. Dotted line represents the limit between low (LFA) and high wild fruit availability (HFA).

During our study period extending over 37 months (34 months of data), edible maize for chimpanzees was available at the border for 22 cumulative months (Figure 2). Regarding wild food resources, half of the months (17 months) were classified as high fruit availability (HFA), with other half classified as low fruit availability (LFA). No significant difference was found in wild food availability between maize seasons (t_26.1_ = −0.269, *p*-value = 0.790), and edible maize was available during both low and high availability of wild fruits.

### 3.2. Focal Nest-To-Nest Distribution

Representing over 3407 h of observation, 640 FNNs were collected (mean duration per FNN = 5 h 17 min; range = 4 min–12 h 27 min) for 27 individuals (12 females and 15 males). The 206 FNNs with a duration superior or equal to 6 h included the monitoring of 20 individuals (three non-maternal adult females (AF), five mothers (MO), nine adult males (AM), and three subadult males (YO); 1 to 35 FNNs per individual, median = 8.5). The distribution of FNNs according to domestic and wild resources is detailed in Table 3.

### 3.3. Diet Composition

Between January 2016 and January 2019, chimpanzees consumed ripe and unripe fruits from 34 species, accounting for an average of 82.7% of their diet and including 11 fig species (56.5%). This proportion of wild fruits appeared to be stable among maize seasons (χ^2^ = 4.97 × 10^−31^, *p* = 1) and wild fruit availability (χ^2^ = 1.41, *p* = 0.495). Leaves (7.3%), pith and stem (1.1%), meat (0.5%), honey (0.3%), flowers, soil, caterpillars, and barks (less than 0.1% each) constituted the rest of the diet, in addition to unspecified THV items (7.4%).

The 13 fruits used for nutritional analysis accounted for almost 70% of the total feeding time and at least 84% of the wild fruit feeding time. They belonged to 9 species, including 6 species of figs, among the 10 most consumed by Sebitoli chimpanzees during our 37-month study. The nutritional analysis results, detailed in Table 2, revealed a mean energy gain of 4.53 kcal·g^−1^ of dry matter, with close values among fruit species (range = 4.23–5.10).

### 3.4. Energy Intakes

**Figure 3:**
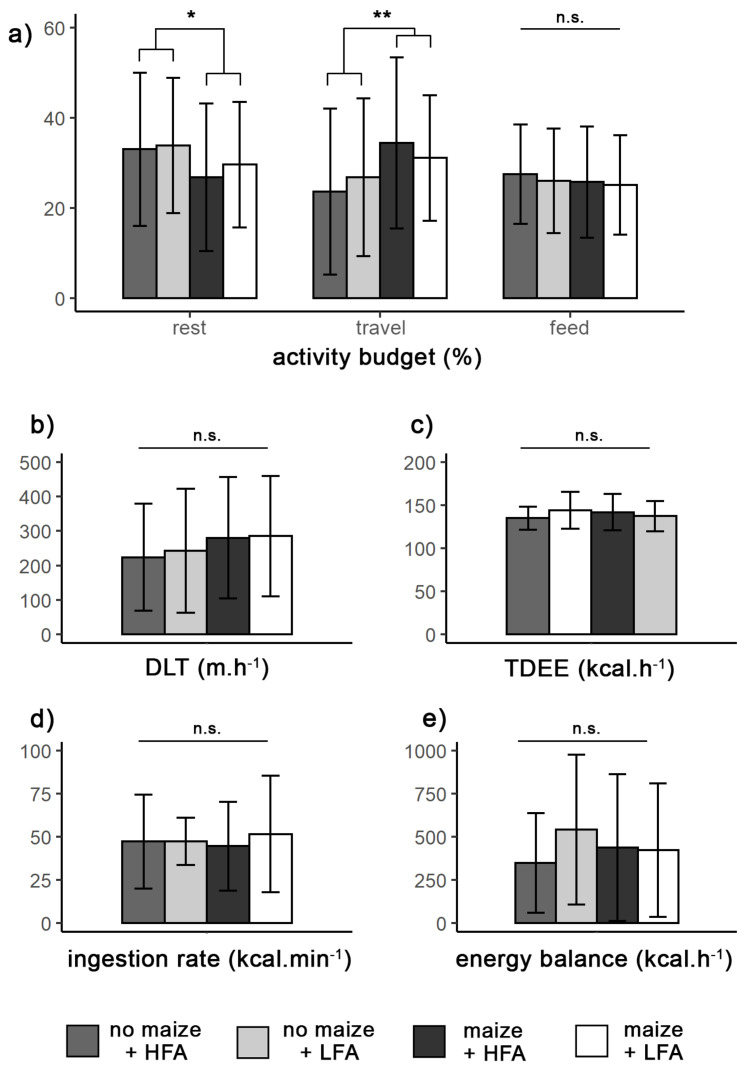
Budget of the main activities (**a**), daily paths (DLT) (**b**), energy expenditures (TDEE) (**c**), ingestion rate (**d**), and energy balance (**e**) of the Sebitoli community according to maize and wild fruit availability between January 2016 and January 2019. LFA: low wild fruit availability; HFA: high wild fruit availability; * *p* < 0.05; ** *p* < 0.005; n.s.: not significant. Bars represent standard deviation.

These 13 fruits represented at least 80% of the feeding time for 112 FNNs. Chimpanzees ingested a constant energy rate from wild fruits, regardless of the maize availability (maize: 48 ± 29.9 kcal·min^−1^ vs. no maize: 47.3 ± 18 kcal·min^−1^; *Q* = 0.002, *p* = 0.965) or the wild fruit availability (HFA: 45.4 ± 26 kcal·min^−1^ vs. LFA: 49.4 ± 25 kcal·min^−1^; *Q* = 2.011, *p* = 0.163), and no significant interaction was noticed between seasons (*Q* = 0.059, *p* = 0.810) (Table 4, Figure 3d).

### 3.5. Activity Budgets, Daily Paths, and Energy Expenditures

The Sebitoli community exhibited different activity budgets depending on maize availability (Pillai’s Trace = 0.043, *F*_3,187_ = 2.80, *p* <0.05). In contrast, wild FAI did not seem to influence the time spent for the different activities (Pillai’s Trace = 0.005, *F*_3,187_ = 0.307, *p* = 0.82). During maize season, chimpanzees increased their traveling time (32.6% vs. 26.2%, *F*_1,189_ = 7.934, *p* <0.01) and reduced their resting time compared to non-maize season (28.5% vs. 34.2%, *F*_1,189_ = 5.238, *p* <0.05), whereas the feeding time in the forest seemed constant in the activity budget, independent of maize availability (25.8% vs. 25.2%, *F*_1,189_ = 0.500, *p* = 0.480). No significant interaction was noticed between maize and wild fruit availability in the activity budget (Pillai’s Trace = 0.014, *F*_3,187_ = 0.863, *p* = 0.46) (Table 5, Figure 3a).

As a result of longer traveling time, the daily path length tended to increase when maize was available (283 vs. 235 m·h^−1^); however, this difference was not significant (*F*_1,189_ = 3.667, *p* = 0.057). Wild fruit availability did not significantly influence the daily paths either (*F*_1,189_ = 0.166, *p* = 0.684). Similar daily expenditures were noticed between maize seasons (*F*_1,187_ = 0.706, *p* = 0.402) and between FAI periods (*F*_1,187_ = 0.214, *p* = 0.644) (Table 5, Figure 3b).

### 3.6. Energy Balance

Only 11 out of 112 FNNs had negative energy balances, 10 of which were during the maize season, 6 during LFA, and 4 during HFA. On average, energy balances seemed to be particularly higher when domestic resources were not available and wild resources were low (no maize * LFA: 541.5 ± 433.9 kcal·h^−1^) (Figure 3e), although the interaction was not significant (*Q* = 0.549, *p* = 0.462). No difference was found according to the availability of maize or wild fruits separately (maize: *Q* = 0.005, *p* = 0.943; FAI: *Q* = 1.075, *p* = 0.305).

### 3.7. Age-Class Contribution to the Community Energy Balance

**Figure 4:**
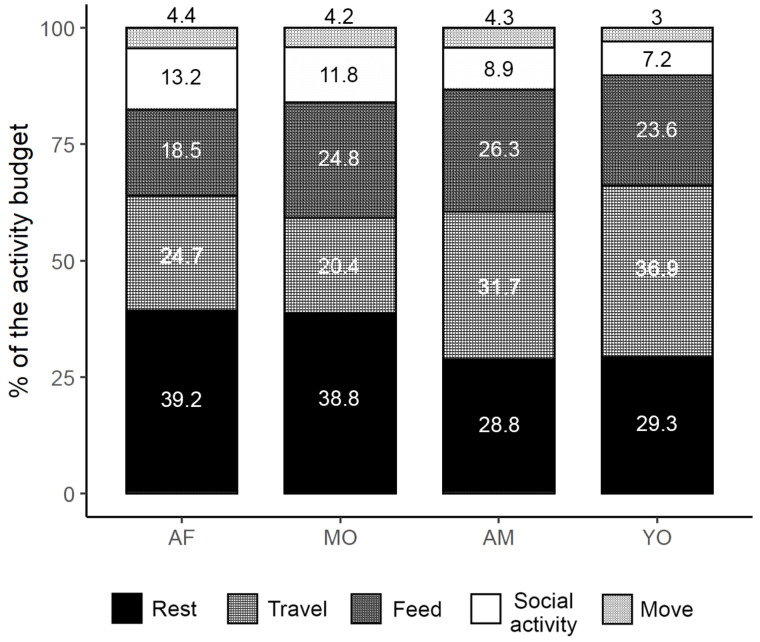
Activity budget of the Sebitoli community according to sex–age classes. AF: non-maternal females; MO: mothers; AM: adult males; YO: subadult males. Values represent the mean value of each activity per class.

Throughout our study period, chimpanzees from the Sebitoli community spent, on average, 31% of their daily time resting, 29.8% traveling, 25.5% feeding, 9.5% socializing, and 4.2% moving in trees (Figure 4). Whereas mothers spent more time resting (H_3_ = 11.6, *p* < 0.01; Dunn’s test, *p* < 0.01) and less time traveling than adult males (% travel: H_3_ = 15.3, *p* < 0.005; Dunn’s test, *p* < 0.005; DLT: H_3_ = 25.4, *p* < 0.0001; Dunn’s test, *p* < 0.001), they had the most important expenditure rate due to lactation and pregnancy costs, with 169 kcal·h^−1^ (SD ± 20.6) (H_3_ = 79.2, *p* < 0.0001). Non-maternal females (TDEE_AF_ ± SD = 114 ± 5.35 kcal·h^−1^) had the lowest energy expenditure (H_3_ = 79.2, *p* <0.0001), also traveling significantly less and resting more than males (Table 6). No significant difference was found for frugivory (H_3_ = 0.22, *p* = 0.974), ingestion rate (H_3_ = 1.8, *p* = 0.614), or energy balance (H_3_ = 1.28, *p* = 0.734) according to sex–age classes (see Supplementary Materials for tests details).

### 3.8. Approximation of Uphill Expenditures

During our study period, chimpanzees’ paths had an average positive slope of 21.6° (SD ± 6.7, range = 0–46.4). Considering a null slope, the Equation from Bobbert (10) provided mean travel expenditures of 47.9 kcal per FNN (± 31.3, range = 0–140.8), i.e., almost four times lower than Taylor’s Equation (4) (172.8 kcal on average). However, by adding positive slopes in Bobbert’s Equation (10), travel expenditures were multiplied by 2.4 in comparison with the null model, with a mean value of 107.6 kcal per FNN (± 69.8, range = 0–292.3). Then, if we apply this ratio to travel expenditures from Taylor’s Equation (4), TDEE increased by 18%, and energy balance decreased by 32% on average (mean = 430.8 kcal·h^−1^, range: −125.4–2086.9).

## 4. Discussion

During our three-year-long study, we analyzed 206 FNNs from 20 chimpanzees to assess the variations in activity budget, daily paths, daily expenditures, intakes from wild fruits, and energy balance according to maize and wild fruit availability at the edge and into Kibale National Park. First, wild fruits are not less available during maize season, and thus, maize does not represent an alternative resource that chimpanzees would use to compensate for wild food shortages. We might expect that, attracted by the convenient and rich resources of the gardens adjacent to their home range, chimpanzees would decrease their activities in the forest and remain at the edge, thereby reducing their travel time. On the contrary, they seem to use the additional energy from maize to increase travel time at the expense of rest but without impacting daily paths and energy expenditures. Finally, regardless of wild food availability, the proportion of wild fruits in the chimpanzee diet and the resulting energy intakes are unchanged throughout the year (Table 7). Our results revealed less variation than expected due to the frequent visits of Sebitoli chimpanzees to the gardens and their significant consumption of maize observed through camera traps [43,55] (SCP unpublished data).

### 4.1. Distance Traveled and Activity Budget

On a 12 h daily basis, feeding usually represents the main activity in the wild chimpanzee activity budget [16,76,96,97,98]. With less time allocated to feed (25.5%) than rest (31%) and travel (29.8%), we found an activity budget similar to that of females from the Mahale Mountains’ M group, Tanzania [99], and, surprisingly, for the *P. troglodytes* community in the Mefou Sanctuary, Cameroon [100]. Due to high slopes, swamps, and dense vegetation in the study site, we suggest a low travel speed by chimpanzees or an overestimation of the travel time by the observers at the cost of other activities (feed, rest, or grooming) on the ground. However, we noticed an important daily range for FNNs of 10 h and more, i.e., FNNs approximating a real nest-to-nest monitoring. Males from the Sebitoli community traveled, on average, 3.4 km per day (*n* = 33), and mothers traveled an average of 2.6 km per day (*n* = 5). This is longer than in the Kanyawara community (2.4 km for males, 2 km for females) [53], which has a similar habitat [60], but lower than in the Mahale Mountains community (range = 2.2–6.4 km) [101].

With such an activity budget and daily lengths, we might expect large energy expenditures. However, TDEE from FNNs of 10 h and greater are close to theoretical values based on Key and Ross’ equation [18,53] for adult males (this study = 1491 kcal vs. theoretical = 1558 kcal) and mothers (this study = 1890 kcal vs. theoretical = 1814 kcal). However, we found larger expenditures for younger chimpanzees (this study = 1357 kcal vs. theoretical = 711 kcal).

### 4.2. The Sebitoli Area: A High-Quality Fruit Habitat

The analysis of the community diet revealed a larger fruit portion (82.7%) than that of other communities from the Kibale National Park (Ngogo: 80.5%, Kanyawara: 64.4%) [102]. In Sebitoli, this frugivory did not differ among seasons (44% of FNN presented a full-fruit diet during LFA months), and we suggest that the wild fruit availability in the forest was sufficient to avoid inducing a change in behavior that would require adjustments in energy expenditures or diet. This assumption is supported by a rotation observed in the species consumed throughout the year. Indeed, if *F. sur* or *F. exasperata* were eaten at least 60% of the study period, other fruits presented a strong seasonality, such as *F. natalensis,* eaten only between January and March; *Drypetes* sp., eaten only from June to August; and *M. bagshawei*, eaten only from October to December. Fig species seem particularly important for Sebitoli chimpanzees (56.5% of the total diet and 6 species among the 10 most consumed) and can be considered as a staple food based on their abundance in the community’s home range and fig trees’ large basal areas [60]. In addition, nutritional analysis from 2015 attested that fruits from the Sebitoli area were bigger, with +16% dry mass in comparison with Kanyawara data [103] and 32% more energy than other Ugandan sites with constant gains between species (Table 2). Thus, these high-caloric and abundant fruits allowed chimpanzees to have high intakes (on average, 47.7 kcal·min^−1^ for 4645 kcal·day^−1^) and thus to maintain positive balances all year long and possibly gain weight.

By focusing the analyses only on FNNs with a high frugivory rate, our results could have been overestimated. Thus, further nutritional analyses on a larger number of fruit species and non-fruit resources are required to complete this study in order to obtain a more accurate overview of Sebitoli chimpanzees’ energy intakes and balance. Future work should focus particularly on *Drypetes* sp., which is rarely mentioned and studied in the primate literature [104,105,106] and the fruits of which represent more than 20% of the frugivory rate observed here. A survey focusing on the availability of preferred and staple food could also reveal different seasonal variations in the behavior of the Sebitoli community.

### 4.3. Maize: The Opportunistic Consumption of an Extra Food

As expected, our study confirmed that when edible maize is available at the forest edges, both ripe and unripe fruits are also available. Maize consumption does not fit with the fallback food concept, instead representing an opportunistic way to gain energy. Similar results were found by Naughton-Treves [107] around KNP and by Hockings et al. [30] in a forest–farm matrix in Bossou, Guinea.

The metabolizable energy of maize seems lower or equivalent to that of wild fruits from our study area (maize: 277–447 kcal/100 g, [108,109]; wild fruits: 423–510 kcal/100 g). However, each maize cob is heavier than fig fruits (cob: 130–179 gDM [110]; dry fig: 0.22–7.14 gDM, Supplementary Materials, Table S5), and their concentration in gardens should reduce chimpanzee foraging efforts (less harvesting time, no climbing, and no wadging). On the contrary, the activity budget analysis revealed lower rest and higher travel time when edible maize was available at the borders. Such results are consistent with chimpanzees’ budget in Bossou [37]. However, due to the variations in crops distribution in our study site (at the edge of the community home range) and the use of some crops as fallback food in Bossou [30], we expected opposite results. The Sebitoli community could benefit from maize for traveling further, and this would seem to correspond to a greater time spent in the field during the maize season (55% vs. 48%). Nevertheless, without significantly longer daily paths, these observations could suggest that chimpanzees reduce their walking speed during the maize season or that the travel time was overestimated due to the topographic characteristics mentioned above.

The absence of a major impact of maize availability on the diet and activities of individuals monitored in the community could also suggest that chimpanzees’ interest in this resource is quite low. Indeed, maize consumption was observed only in 7 out of 206 FNNs. Nevertheless, in a crop-foraging context, chimpanzees can be vigilant, silent, and anxious [33,43,111] (personal observation). This behavior may hinder chimpanzee monitoring at the forest edge. In addition, field assistants follow strict regulations, stopping monitoring once near the edge to avoid the chimpanzees becoming confident in crop foraging, feeling protected by their presence. The Sebitoli chimpanzee community also visits gardens during darkness [43], i.e., after 19:00, when chimpanzee monitoring is usually already over for visibility and safety purposes, sometimes climbing down from the nest to enter the maize gardens. Moreover, camera trap data and farmer interviews during our study period suggest frequent crop foraging by chimpanzees (SCP unpublished data). Thus, we assume that the wild fruits provided by the forest are sufficient all year long, and chimpanzees enjoy maize as an additional food, a bonus in their diet. Furthermore, this combination of highly nutritious and abundant wild and domestic resources could explain the high density of chimpanzees in the Sebitoli area (4.1 ind./km^2^) [60].

Camera traps could provide additional information on crop-foraging behavior, such as identification of individuals coming into maize gardens or an estimated duration of crop foraging events or nesting delay. However, the lack of visibility at night (even with a day/night sensor) may bias these data or limit their acquisition.

### 4.4. A Preliminary Study about An Energy-Balance Proxy

Due to the methodology used and missing nutrition data, our results must be examined as a proxy of energy balance to assess the health status of the Sebitoli chimpanzee community. Indeed, the factorial approaches—based on BMR, activity budgets, and mean body mass—underestimate real expenditures by only considering them as a simple additive equation and denying a more complex link between energy balance and genetics or population environment [112,113]. Nevertheless, avoiding such approximation for free-ranging populations remains a major challenge. More precise methods based on double-labeled water [113] or respirometry [114] require strictly controlled conditions with stressful situations (individual capture) and material supply—mainly reproducible in a captive context, in sanctuary, or on small-size species. Recently, estimating the concentration of urinary C-peptide, a biomarker of insulin secretion [115], appeared to be an efficient alternative and a non-invasive method to evaluate energy balance and its seasonal variations for primates [52,116,117]. However, this method requires shipment and process constraints [118], which are not always applicable at every study site.

### 4.5. Uphill Expenditure Approximation

Because grades and substrate firmness were not included in our analysis, we postulate that we might have underestimated traveling costs and expenditures. The Sebitoli area has steep slopes and grades (up to 50%, SCP unpublished data), with wetlands at the bottom of valleys. Uphill walking, along with downhill walking with a 20% or more negative grade [119], modified speed [120] and oxygen consumption [115,116,121,122] and enhanced expenditures. By simply including ascending elevations [82] in our data, the cost of travel could double and decrease energy balance by 32%. Sharper corrections based on slope degrees [122] could also be applied, as well as factors depending on substrate firmness (revised in [123]) and the swamps often crossed by Sebitoli chimpanzees. However, as equations are modeled on non-arboreal bipeds, further research is required before using it on non-human primates.

### 4.6. Health Consequences and Conservation Issues

Less than 10% of FNNs revealed a negative balance with an insufficient ingestion rate. These observations concerned female and male chimpanzee, maize and non-maize seasons, and HLA and LFA months. Consequently, the monitored individuals seemed to be healthy from a strictly energy point of view, using maize as a bonus treat in addition to wild fruits. Such a diet raises questions about long-term health consequences, especially since maize availability is increasing at the western border as farmers recently began to cultivate and harvest it year-round (Krief personal comment). In some extreme cases, the consumption of items with a high energetic value, such as crops, could lead to physiologic consequences, such as earlier sexual maturity; increased fatness and body mass [124]; and pathologic developments, such as obesity and/or diabetes [125].

Moreover, garden proximity exposes chimpanzees to threats, such as snares, spears, or dog attacks [49], as well as chemicals spread in the surrounding plantations and fields. A cocktail of 13 pesticides with in vivo disruptive effects on the thyroid–estrogen axis of *Xenopus laevis* were found in maize seeds and stems, as well as in rivers and swamps through the Sebitoli area [126,127].

## 5. Conclusions

Overall, this study sheds new light on the motivation of the Sebitoli community for crop-foraging at the edge of a high-quality habitat. Similarly to other frugivorous primates and mammals, chimpanzees play an essential ecological role in seed dispersal and the regeneration and the structure of forest habitats [8,128,129]. Thus, changing their diet by substituting wild fruits with domestic crops could threaten this balance. Despite the availability of convenient and nutritious resources at their territory edge, Sebitoli chimpanzees still exploit wild resources and do not limit their movements. This suggests that their contribution to sustaining the forest in this area would not be affected. Additionally, these results must be taken into consideration for organizing and implementing local mitigation measures to prevent chimpanzees’ incursions in gardens and reduce associated risks for human populations and for wildlife. Since the forest appears to provide sufficient resources and maize is not an essential fallback food for the survival of Sebitoli chimpanzees, we recommend the installation of buffer zones with unpalatable crops between the fields and the forest. Although the majority of great apes live outside of protected areas where human activities are expanding [130], improving human–ape cohabitation at the border of preserved habitats is essential for the conservation of these threatened species.

## Figures and Tables

**Table 1:** Ethogram of the behavior categories used in the evaluation of chimpanzees’ energy balance in Kibale National Park, Uganda.

Category	Code	Description
Feeding	F	Ingestion of a food item (including wadging)
Resting	R	Prolonged motionless and inactive state, sit or laid down
Traveling	T	Locomotion on the ground from one point to another (excluding displaying and chasing)
Moving in trees	M	Locomotion in a tree, vertically or horizontally (including to forage or socialize)
Social activities	SA	Interaction with another individual or self-grooming
High social activities	HSA	Interaction involving locomotion or important body movements (copulating, displaying, chasing, playing)
Low social activities	LSA	Motionless interaction (including grooming, self-grooming, vocalizing, etc.)

**Table 2:** Energy concentration (kcal/gDM) in 13 fruits from Sebitoli and intersite variations across Uganda.

Species	Part	Kibale National Park							
		SBL ^1^		KNP ^2^	KWRA.1 ^3^	KWRA.2 ^4^	KWRA.3 ^5^	NGO ^6^	Bulindi ^7^
		N	X (SD)						
*Aphania senegalensis*	RF	3	4.23 (0.07)	-	-	-	-	-	-
*Cordia abyssinica*	RF	2	4.42 (0.55)	-	-	-	3.28	-	-
*Ficus sansibarica*	RF	3	4.75 (0.68)	4.64	2.20	3.05	3.28	2.96	3.36
*Ficus saussureana*	RF	5	4.60 (0.38)	4.47	-	-	-	-	-
*Ficus exasperata*	RF	2	4.23 (0.16)	5.03	-	2.90	-	-	3.45
	URF	1	5.10 (-)	-	1.90	2.99	-	-	-
*Ficus mucoso*	RF	2	4.53 (0.08)	-	2.40	-	-	2.83	3.56
*Ficus natalensis*	RF	6	4.49 (0.05)	4.37	1.30	-	2.93	-	3.23
	URF	-	4.49 *	-	-	-	2.56	-	-
*Ficus sur*	RF	24	4.53 (0.69)	4.53	-	2.55	3.13	2.60	3.41
	URF	5	4.41 (0.38)	-	-	-	2.80	-	-
*Mimusops bagshawei*	RF	-	4.50 *	-	-	2.93	3.03	2.89	-
	URF	2	4.50 (0.09)	-	-	-	2.78	-	-

N: number of samples; X: mean energy gain in kcal/g of dry matter (DM); SD: standard deviation. Values for the different sites are from: ^1^ this study, ^2^ [84], ^3^ [85], ^4^ [86], ^5^ [87], ^6^ [88], and ^7^ [31]. SBL: Sebitoli; KNP: Kibale National Park (no site specified); KWRA: Kanyawara; NGO: Ngogo, all sites are located in Uganda; RF: ripe fruit; URF: unripe fruit; * missing values in our study (we took RF and URF values instead).

**Table 3:** Study design (number of FNNs) according to maize and wild fruit availability.

	All	Maize			No Maize		
		HFA	LFA	na	HFA	LFA	na
All	206	31	51	10	48	63	3
80% *	112	30	29	-	14	33	6

Index of wild fruit availability. HFA: high fruit availability; LFA: low fruit availability for wild fruits; na: months with missing phenology data (*n* = 3); * only FNNs with at least 80% of the total feeding time covered by the 13 fruits used in nutritional analysis.

**Table 4:** Results of the robust two-way ANOVA for ingestion rate, daily paths, energy expenditures, and energy balance.

Response Variable	Dependent Variable	*Q Statistics*	*p*-Value
Ingestion rate	Maize	0.002	0.965
	FAI	2.011	0.163
	Maize * FAI	0.059	0.810
Daily length traveled (DLT)	Maize	2.310	0.133
	FAI	0.015	0.902
	Maize * FAI	0.002	0.968
Energy expenditure (TDEE)	Maize	0.032	0.860
	FAI	0.580	0.449
	Maize * FAI	3.974	0.050
Energy balance	Maize	0.005	0.943
	FAI	1.075	0.305
	Maize * FAI	0.549	0.462

Maize: maize availability, two levels: maize, non-maize; FAI: wild fruit availability, two levels: high (HFA), low (LFA), * defines the interaction between the variables.

**Table 5:** Results of two-way MANOVA and two-way ANOVA for the activity budget.

Two-Way MANOVA		Pillai’s Trace	*F*	n *df*	*df*	*p*-Value
Response Variable	Dependent Variable					
Feed, rest, travel	Maize	0.043	2.799	3	187	0.041 *
	FAI	0.005	0.307	3	187	0.820
	Maize * FAI	0.014	0.863	3	187	0.461
**Two-Way ANOVA (post hoc test)**		**SS**	***F***	**n *df***	***df***	***p*-Value**
**Response Variable**	**Dependent Variable**					
Feed	Maize	66.3	0.500	1	189	0.480
	FAI	44.8	0.338	1	189	0.562
	Maize * FAI	8.5	0.050	1	189	0.800
Rest	Maize	1232	5.238	1	189	0.023 *
	FAI	185	0.786	1	189	0.376
	Maize * FAI	47	0.202	1	189	0.654
Travel	Maize	2278	7.934	1	189	0.005 **
	FAI	18	0.028	1	189	0.562
	Maize * FAI	473	1.649	1	189	0.800

Maize: maize availability, two levels: maize, non-maize; FAI: wild fruit availability, two levels: high (HFA), low (LFA). After *p*-value, * indicates *p*-value < 0.05, ** indicates *p*-value < 0.01

**Table 6:** Contribution of sex–age classes in daily paths, energy balance, and frugivory between January 2016 and January 2019, all seasons included.

Sex-Age Class		All					80% *			
		N_i_	N_FNN_	DLT (m·h^−1^)	TDEE (kcal·h^−1^)	% Frugivory	N_i_	N_FNN_	Ingestion Rate (kcal·min^−1^)	Energy Balance (kcal·h^−1^)
				X (SD)	X (SD)	X (SD)			X (SD)	X (SD)
Females	AF	3	9	175 (102)	114 (5.4)	85.1 (23.7)	3	6	48.1 (20.1)	314.3 (252.6)
	MO	5	36	155 (140)	169 (20.6)	85.5 (20.3)	5	21	49.3 (15.9)	479.1 (425.6)
Males	AM	9	147	299 (172)	135 (10.6)	84.1 (21.8)	8	77	47.4 (27.9)	460.3 (418)
	YO	3	14	241 (144)	133 (8.9)	79.6 (29.5)	3	8	45.5 (18.7)	426.1 (289.6)
All		20	206	264 (172)	140 (19)	84 (22.1)	19	112	47.7 (24.9)	453.5 (402.1)

AF: non-maternal adult females; MO: mothers; AM: adult males; YO: subadult males; N_i_: number of individuals; N_FNN_: number of FNNs superior or equal to 6 h duration; DLT: daily length traveled; TDEE: total daily energy expenditures; X (SD): mean value (standard deviation); * only FNNs with at least 80% of the total feeding time covered by the 13 fruits used in nutritional analysis.

**Table 7:** Verification of the main hypothesis and summary of the results from this study.

Hypothesis: Opportunistic Maize Consumption by Chimpanzees							
Maize	Wild Fruits	Rest	Travel/Daily Paths		Wild Frugivory and Intakes		Energy Expenditures
1	High/low	+	−		−		−
0	High/low	−	+		+		+
**Results: Opportunistic Maize Consumption by Chimpanzees**							
**Maize**	**Wild Fruits**	**Rest (%)**	**Travel (%)**	**Daily Paths (m·h^−1^)**	**Wild** **Frugivory (%)**	**Wild Intake (kcal·min^−1^)**	**Energy Expenditure (kcal·h^−1^)**
1	High/low *	28.5	32.6	282.7	84.2	48.0	139.6
		˄	˅	=	=	=	=
0	High/low **	34.2	26.2	241.5	83.9	47.3	139.8

* For all categories, mean values during the maize season. ** For all categories, mean values during the non-maize season.

## Data Availability

Not applicable.

## Supplementary Materials

The following supporting information can be downloaded at: https://www.mdpi.com/article/10.3390/ani12070806/s1. Table S1: List of chimpanzees monitored by the FNN protocol between January 2016 and January 2019 in Kibale National Park, Uganda. Table S2: Repartition of FNN among sex–age classes, maize seasons, and wild fruit availability between January 2016 and January 2019 in Kibale National Park, Uganda. Table S3: Detailed statistics between sex–age classes in activity budget, daily paths, energy expenditures, frugivory, energy intakes, and energy balance. Text S1: Nutritional analysis protocol. Table S4: List of wild fruits consumed by the Sebitoli community between January 2016 and January 2019 in Kibale National Park, Uganda. Table S5: Dry mass (g) of 13 fruits from Sebitoli and Kanyawara in Kibale National Park, Uganda.

## References

[1] Smith-Gill S.J. (1975). Cytophysiological Basis of Disruptive Pigmentary Patterns in the Leopard FrogRana Pipiens. II. Wild Type and Mutant Cell-Specific Patterns. J. Morphol..

[2] Hubbell S.P. (1979). Tree Dispersion, Abundance, and Diversity in a Tropical Dry Forest: That Tropical Trees Are Clumped, Not Spaced, Alters Conceptions of the Organization and Dynamics. Science.

[3] Hladik C.M., Tuttle R.H. (1975). Ecology, Diet, and Social Patterns in Old World and New World Primates. Socioecology and Psychology of Primates.

[4] Chapman C.A., Wrangham R.W., Chapman L.J., Kennard D.K., Zanne A.E. (1999). Fruit and Flower Phenology at Two Sites in Kibale National Park, Uganda. J. Trop. Ecol..

[5] Janmaat K.R.L., Ban S.D., Boesch C. (2013). Chimpanzees Use Long-Term Spatial Memory to Monitor Large Fruit Trees and Remember Feeding Experiences across Seasons. Anim. Behav..

[6] Trapanese C., Meunier H., Masi S. (2019). What, Where and When: Spatial Foraging Decisions in Primates: Spatial Foraging Decisions in Primates. Biol. Rev..

[7] Lambert J.E., Garber P.A. (1998). Evolutionary and Ecological Implications of Primate Seed Dispersal. Am. J. Primatol..

[8] Stoner K.E., Riba-Hernández P., Vulinec K., Lambert J.E. (2007). The Role of Mammals in Creating and Modifying Seedshadows in Tropical Forests and Some Possible Consequences of Their Elimination. Biotropica.

[9] Malenky R.K., Wrangham R.W. (1994). A Quantitative Comparison of Terrestrial Herbaceous Food Consumption by *Pan paniscus* in the Lomako Forest, Zaire, and *Pan troglodytes* in the Kibale Forest, Uganda. Am. J. Primatol..

[10] Chapman C.A., Chapman L.J., Wrangham R.W. (1995). Ecological Constraints on Group Size: An Analysis of Spider Monkey and Chimpanzee Subgroups. Behav. Ecol. Sociobiol..

[11] Wrangham R.W. (2000). Why Are Male Chimpanzees More Gregarious than Mothers? A Scramble Competition Hypothesis. Primate Males—Causes and Consequences of Variation in Group Composition.

[12] Chancellor R.L., Rundus A.S., Nyandwi S. (2012). The Influence of Seasonal Variation on Chimpanzee (*Pan troglodytes schweinfurthii*) Fallback Food Consumption, Nest Group Size, and Habitat Use in Gishwati, a Montane Rain Forest Fragment in Rwanda. Int. J. Primatol..

[13] Marshall A.J., Wrangham R.W. (2007). Evolutionary Consequences of Fallback Foods. Int. J. Primatol..

[14] Knott C.D. (1998). Changes in Orangutan Caloric Intake, Energy Balance, and Ketones in Response to Fluctuating Fruit Availability. Int. J. Primatol..

[15] Stevenson P.R., Quinones M.J., Ahumada J.A. (2000). Influence of Fruit Availability on Ecological Overlap among Four Neotropical Primates at Tinigua National Park, Colombia1. Biotropica.

[16] Doran D. (1997). Influence of Seasonality on Activity Patterns, Feeding Behavior, Ranging, and Grouping Patterns in Taï Chimpanzees. Int. J. Primatol..

[17] Dunn J.C., Cristóbal-Azkarate J., Veà J.J. (2009). Differences in Diet and Activity Pattern between Two Groups of *Alouatta Palliata* Associated with the Availability of Big Trees and Fruit of Top Food Taxa. Am. J. Primatol..

[18] Key C., Ross C. (1999). Sex Differences in Energy Expenditure in Non–Human Primates. Proc. R. Soc. Lond. B Biol. Sci..

[19] Wright E., Robbins A.M., Robbins M.M. (2014). Dominance Rank Differences in the Energy Intake and Expenditure of Female Bwindi Mountain Gorillas. Behav. Ecol. Sociobiol..

[20] Thompson M.E., Machanda Z.P., Fox S.A., Sabbi K.H., Otali E., Thompson González N., Muller M.N., Wrangham R.W. (2020). Evaluating the Impact of Physical Frailty during Ageing in Wild Chimpanzees (*Pan troglodytes schweinfurthii*). Philos. Trans. R. Soc. B Biol. Sci..

[21] Estrada A. (2013). Socioeconomic Contexts of Primate Conservation: Population, Poverty, Global Economic Demands, and Sustainable Land Use: Social Contexts of Primate Conservation. Am. J. Primatol..

[22] Estrada A., Garber P.A., Rylands A.B., Roos C., Fernandez-Duque E., Di Fiore A., Nekaris K.A.-I., Nijman V., Heymann E.W., Lambert J.E. (2017). Impending Extinction Crisis of the World’s Primates: Why Primates Matter. Sci. Adv..

[23] Rijksen H.D., Meijaard E. (1999). Our Vanishing Relative. The Status of Wild Orangutans at the Close of the Twentieth Century.

[24] Hockings K., Humley T. (2009). Best Practice Guidelines for the Prevention and Mitigation of Conflict between Humans and Great Apes.

[25] Great Apes Survival Partnership, International Union for Conservation of Nature (2018). Report to the CITES Standing Committee on the Status of Great Apes.

[26] Carvalho J.S., Graham B., Bocksberger G., Maisels F., Williamson E.A., Wich S., Sop T., Amarasekaran B., Barca B., Barrie A. (2021). Predicting Range Shifts of African Apes under Global Change Scenarios. Divers. Distrib..

[27] International Union for Conservation of Nature (2021). The IUCN Red List of Threatened Species. https://www.iucnredlist.org/.

[28] Thompson M.E., Sabbi K.H. Evolutionary Demography of the Great Apes; 2019. https://osf.io/r9pz7/.

[29] Galdikas B.M.F., Wood J.W. (1990). Birth Spacing Patterns in Humans and Apes. Am. J. Phys. Anthropol..

[30] Hockings K.J., Anderson J.R., Matsuzawa T. (2009). Use of Wild and Cultivated Foods by Chimpanzees at Bossou, Republic of Guinea: Feeding Dynamics in a Human-Influenced Environment. Am. J. Primatol..

[31] McLennan M.R., Ganzhorn J.U. (2017). Nutritional Characteristics of Wild and Cultivated Foods for Chimpanzees (*Pan troglodytes*) in Agricultural Landscapes. Int. J. Primatol..

[32] Hill C.M. (2005). People, Crops and Primates: A Conflict of Interests. Commensalism and Conflict: The Human–Primate Interface.

[33] Hockings K.J., Humle T., Anderson J.R., Biro D., Sousa C., Ohashi G., Matsuzawa T. (2007). Chimpanzees Share Forbidden Fruit. PLoS ONE.

[34] Carlitz E.H.D., Miller R., Kirschbaum C., Gao W., Hänni D.C., van Schaik C.P. (2016). Measuring Hair Cortisol Concentrations to Assess the Effect of Anthropogenic Impacts on Wild Chimpanzees (*Pan troglodytes*). PLoS ONE.

[35] Naughton-Treves L. (1997). Farming the Forest Edge: Vulnerable Places and People around Kibale National Park, Uganda. Geogr. Rev..

[36] Osborn F.V., Hill C.M., Woodroffe R., Thirgood S., Rabinowitz A. (2005). Techniques to Reduce Crop Loss: Human and Technical Dimensions in Africa. People and Wildlife.

[37] Hockings K.J., Anderson J.R., Matsuzawa T. (2012). Socioecological Adaptations by Chimpanzees, *Pan troglodytes verus*, Inhabiting an Anthropogenically Impacted Habitat. Anim. Behav..

[38] Campbell-Smith G., Campbell-Smith M., Singleton I., Linkie M. (2011). Raiders of the Lost Bark: Orangutan Foraging Strategies in a Degraded Landscape. PLoS ONE.

[39] Saj T., Sicotte P., Paterson J.D. (1999). Influence of Human Food Consumption on the Time Budget of Vervets. Int. J. Primatol..

[40] Cancelliere E.C., Chapman C.A., Twinomugisha D., Rothman J.M. (2018). The Nutritional Value of Feeding on Crops: Diets of Vervet Monkeys in a Humanized Landscape. Afr. J. Ecol..

[41] Dhawale A.K., Kumar M.A., Sinha A. (2020). Changing Ecologies, Shifting Behaviours: Behavioural Responses of a Rainforest Primate, the Lion-Tailed Macaque *Macaca silenus*, to a Matrix of Anthropogenic Habitats in Southern India. PLoS ONE.

[42] Altmann J., Muruthi P. (1988). Differences in Daily Life between Semiprovisioned and Wild-Feeding Baboons. Am. J. Primatol..

[43] Krief S., Cibot M., Bortolamiol S., Seguya A., Krief J.-M., Masi S. (2014). Wild Chimpanzees on the Edge: Nocturnal Activities in Croplands. PLoS ONE.

[44] Plumptre A.J., Hart J.A., Hicks T.C., Nixon S., Piel A.K., Pintea L. (2016). *Pan Troglodytes* Ssp. *Schweinfurthii*. The IUCN Red List of Threatened Species.

[45] Kalbitzer U., Chapman C.A., Kalbitzer U., Jack K.M. (2018). Primate Responses to Changing Environments in the Anthropocene. Primate Life Histories, Sex Roles, and Adaptability.

[46] Hill C.M. (2017). Primate Crop Feeding Behavior, Crop Protection, and Conservation. Int. J. Primatol..

[47] McLennan M.R., Spagnoletti N., Hockings K.J. (2017). The Implications of Primate Behavioral Flexibility for Sustainable Human–Primate Coexistence in Anthropogenic Habitats. Int. J. Primatol..

[48] McLennan M.R., Hyeroba D., Asiimwe C., Reynolds V., Wallis J. (2012). Chimpanzees in Mantraps: Lethal Crop Protection and Conservation in Uganda. Oryx.

[49] Cibot M., Roux S.L., Rohen J., McLennan M.R. (2019). Death of a Trapped Chimpanzee: Survival and Conservation of Great Apes in Unprotected Agricultural Areas of Uganda. Afr. Primates.

[50] Fedigan L.M. (2010). Ethical Issues Faced by Field Primatologists: Asking the Relevant Questions. Am. J. Primatol..

[51] Cunningham E.P., Unwin S., Setchell J.M. (2015). Darting Primates in the Field: A Review of Reporting Trends and a Survey of Practices and Their Effect on the Primates Involved. Int. J. Primatol..

[52] Behringer V., Deschner T. (2017). Non-Invasive Monitoring of Physiological Markers in Primates. Horm. Behav..

[53] Pontzer H., Wrangham R.W. (2004). Climbing and the Daily Energy Cost of Locomotion in Wild Chimpanzees: Implications for Hominoid Locomotor Evolution. J. Hum. Evol..

[54] N’guessan A.K., Ortmann S., Boesch C. (2009). Daily Energy Balance and Protein Gain Among *Pan troglodytes verus* in the Taï National Park, Côte d’Ivoire. Int. J. Primatol..

[55] Bortolamiol S., Cohen M., Jiguet F., Pennec F., Seguya A., Krief S. (2016). Chimpanzee Non-Avoidance of Hyper-Proximity to Humans. J. Wildl. Manag..

[56] Naughton-Treves L., Treves A., Chapman C., Wrangham R. (1998). Temporal Patterns of Crop-Raiding by Primates: Linking Food Availability in Croplands and Adjacent Forest. J. Appl. Ecol..

[57] Plumptre A.J., Williamson E.A., Rose R., Nangendo G., Didier K., Hart J., Mulindahabi F. (2010). Eastern Chimpanzee (Pan troglodytes schweinfurthii): Status Survey and Conservation Action Plan 2010–2020.

[58] Hartter J.N. (2007). Landscape Change around Kibale National Park, Uganda: Impacts on Land Cover, Land Use, and Livelihoods. Ph.D. Thesis.

[59] Struhsaker T.T. (1997). Ecology of an African Rain Forest: Logging in Kibale and the Conflict between Conservation and Exploitation.

[60] Bortolamiol S., Cohen M., Potts K., Pennec F., Rwaburindore P., Kasenene J., Seguya A., Vignaud Q., Krief S. (2014). Suitable Habitats for Endangered Frugivorous Mammals: Small-Scale Comparison, Regeneration Forest and Chimpanzee Density in Kibale National Park, Uganda. PLoS ONE.

[61] Stampone M.D., Hartter J.N., Chapman C.A., Ryan S.J. (2011). Trends and Variability in Localized Precipitation Around Kibale National Park, Uganda, Africa. Res. J. Environ. Earth Sci..

[62] Bortolamiol S., Krief S., Jiguet F., Palibrk M., Rwaburindore P., Kasenene J., Seguya A., Cohen M. (2013). Analyse Spatiale Des Facteurs Influençant La Répartition Des Chimpanzés à Sebitoli, Parc National de Kibale. Cart. Géomat..

[63] Krief S., Iglesias-González A., Appenzeller B.M.R., Okimat J.P., Fini J.-B., Demeneix B., Vaslin-Reimann S., Lardy-Fontan S., Guma N., Spirhanzlova P. (2020). Road Impact in a Protected Area with Rich Biodiversity: The Case of the Sebitoli Road in Kibale National Park, Uganda. Environ. Sci. Pollut. Res..

[64] Pontzer H., Wrangham R.W. (2006). Ontogeny of Ranging in Wild Chimpanzees. Int. J. Primatol..

[65] Cibot M., Krief S., Philippon J., Couchoud P., Seguya A., Pouydebat E. (2016). Feeding Consequences of Hand and Foot Disability in Wild Adult Chimpanzees (*Pan troglodytes schweinfurthii*). Int. J. Primatol..

[66] Nissen H.W., Yerkes R.M. (1943). Reproduction in the Chimpanzee: Report on Forty-Nine Births. Anat. Rec..

[67] Wallis J. (1997). A Survey of Reproductive Parameters in the Free-Ranging Chimpanzees of Gombe National Park. Reproduction.

[68] Hasegawa T., Hiraiwa-Hasegawa M. (1983). Opportunistic and Restrictive Matings among Wild Chimpanzees in the Mahale Mountains, Tanzania. J. Ethol..

[69] Thompson M.E. (2013). Reproductive Ecology of Female Chimpanzees: Chimpanzee Reproductive Ecology. Am. J. Primatol..

[70] Potts K.B., Chapman C.A., Lwanga J.S. (2009). Floristic Heterogeneity between Forested Sites in Kibale National Park, Uganda: Insights into the Fine-Scale Determinants of Density in a Large-Bodied Frugivorous Primate. J. Anim. Ecol..

[71] McLennan M.R. (2013). Diet and Feeding Ecology of Chimpanzees (*Pan troglodytes*) in Bulindi, Uganda: Foraging Strategies at the Forest–Farm Interface. Int. J. Primatol..

[72] Altmann J. (1974). Observational Study of Behavior: Sampling Methods. Behaviour.

[73] Robert-McComb J.J., Carnero E.Á., Iglesias-Gutiérrez E., Robert-McComb J.J., Norman R.L., Zumwalt M. (2014). Estimating Energy Requirements. The Active Female: Health Issues Throughout the Lifespan.

[74] Kleiber M. (1961). The Fire of Life. An Introduction to Animal Energetics.

[75] Smith R.J., Jungers W.L. (1997). Body Mass in Comparative Primatology. J. Hum. Evol..

[76] Leonard W.R., Robertson M.L. (1997). Comparative Primate Energetics and Hominid Evolution. Am. J. Phys. Anthropol..

[77] Bergstrom M.L., Kalbitzer U., Campos F.A., Melin A.D., Emery Thompson M., Fedigan L.M. (2020). Non-Invasive Estimation of the Costs of Feeding Competition in a Neotropical Primate. Horm. Behav..

[78] Taylor C.R., Heglund N.C. (1982). Energetics and Mechanics of Terrestrial Locomotion. Annu. Rev. Physiol..

[79] Hunt K.D. (1992). Positional Behavior of *Pan troglodytes* in the Mahale Mountains and Gombe Stream National Parks, Tanzania. Am. J. Phys. Anthropol..

[80] Morbeck M.E., Zihlman A.L. (1989). Body Size and Proportions in Chimpanzees, with Special Reference to *Pan troglodytes schweinfurthii* from Gombe National Park, Tanzania. Primates.

[81] Mermier C.M., Robergs R.A., McMinn S.M., Heyward V.H. (1997). Energy Expenditure and Physiological Responses during Indoor Rock Climbing. Br. J. Sports Med..

[82] Bobbert A.C. (1960). Energy Expenditure in Level and Grade Walking. J. Appl. Physiol..

[83] Rothman J.M., Chapman C.A., Van Soest P.J. (2012). Methods in Primate Nutritional Ecology: A User’s Guide. Int. J. Primatol..

[84] Hendrick E.L., Shipley L.A., Hagerman A.E., Kelley L.M. (2009). Fruit and Fibre: The Nutritional Value of Figs for a Small Tropical Ruminant, the Blue Duiker (*Cephalophus monticola*). Afr. J. Ecol..

[85] Conklin N.L., Wrangham R.W. (1994). The Value of Figs to a Hind-Gut Fermenting Frugivore: A Nutritional Analysis. Biochem. Syst. Ecol..

[86] Conklin-Brittain N., Knott C., Wrangham R.W. (2006). Energy Intake by Wild Chimpanzees and Orangutans: Methodological Considerations and a Preliminary Comparison. Feeding Ecology in Apes and Other Primates: Ecological, Physical, and Behavioral Aspects.

[87] Houle A., Wrangham R.W. (2021). Contest Competition for Fruit and Space among Wild Chimpanzees in Relation to the Vertical Stratification of Metabolizable Energy. Anim. Behav..

[88] Potts K.B., Baken E., Ortmann S., Watts D.P., Wrangham R.W. (2015). Variability in Population Density Is Paralleled by Large Differences in Foraging Efficiency in Chimpanzees (*Pan troglodytes*). Int. J. Primatol..

[89] Israel G.D. (1992). Determining Sample Size.

[90] Kwak S.G., Kim J.H. (2017). Central Limit Theorem: The Cornerstone of Modern Statistics. Korean J. Anesthesiol..

[91] Scheiner S.M., Gurevitch J. (2001). Design and Analysis of Ecological Experiments.

[92] Wilcox R.R. (2012). Introduction to Robust Estimation and Hypothesis Testing (Statistical Modeling and Decision Science).

[93] Mair P., Wilcox R. (2020). Robust Statistical Methods Using WRS2. Behav. Res. Methods.

[94] Mair P., Wilcox R., Patil I., Mair M.P. (2021). Package ‘WRS2’. https://cran.fhcrc.org/web/packages/WRS2/WRS2.pdf.

[95] R Core Team (2017). R Foundation for Statistical Computing.

[96] Lehmann J., Boesch C. (2004). To Fission or to Fusion: Effects of Community Size on Wild Chimpanzee (*Pan troglodytes verus*) Social Organisation. Behav. Ecol. Sociobiol..

[97] Bates L.A., Byrne R.W. (2009). Sex Differences in the Movement Patterns of Free-Ranging Chimpanzees (*Pan troglodytes schweinfurthii*): Foraging and Border Checking. Behav. Ecol. Sociobiol..

[98] Murray C.M., Lonsdorf E.V., Eberly L.E., Pusey A.E. (2009). Reproductive Energetics in Free-Living Female Chimpanzees (*Pan troglodytes schweinfurthii*). Behav. Ecol..

[99] Matsumoto-Oda A., Oda R. (1999). Changes in the Activity Budget of Cycling Female Chimpanzees. Am. J. Primatol..

[100] Maurice M.E., Gildas O.A.F., Ekale B.N., Fawoh J.J. (2020). The Activity Budget of Adult Chimpanzees (*Pan troglodytes troglodytes*) and Environmental Conditions in Mefou Primate Sanctuary, Centre Region, Cameroon. Asian J. Res. Zool..

[101] Matsumoto-Oda A. (2002). Behavioral Seasonality in Mahale Chimpanzees. Primates.

[102] Potts K.B., Watts D.P., Wrangham R.W. (2011). Comparative Feeding Ecology of Two Communities of Chimpanzees (*Pan troglodytes*) in Kibale National Park, Uganda. Int. J. Primatol..

[103] Houle A., Conklin-Brittain N.L., Wrangham R.W. (2014). Vertical Stratification of the Nutritional Value of Fruit: Macronutrients and Condensed Tannins: Intratree Variations in Macronutrients. Am. J. Primatol..

[104] Conklin-Brittain N.L., Wrangham R.W., Hunt K.D. (1998). Dietary Response of Chimpanzees and Cercopithecines to Seasonal Variation in Fruit Abundance. II. Macronutrients. Int. J. Primatol..

[105] Furuichi T., Hashimoto C., Tashiro Y. (2001). Fruit Availability and Habitat Use by Chimpanzees in the Kalinzu Forest, Uganda: Examination of Fallback Foods. Int. J. Primatol..

[106] Watts D.P., Potts K.B., Lwanga J.S., Mitani J.C. (2012). Diet of Chimpanzees (*Pan troglodytes schweinfurthii*) at Ngogo, Kibale National Park, Uganda, 2. Temporal Variation and Fallback Foods: Chimpanzee Diet Variation at Ngogo. Am. J. Primatol..

[107] Naughton-Treves L. (1998). Predicting Patterns of Crop Damage by Wildlife around Kibale National Park, Uganda. Conserv. Biol..

[108] Bryson-Morrison N., Beer A., Gaspard Soumah A., Matsuzawa T., Humle T. (2020). The Macronutrient Composition of Wild and Cultivated Plant Foods of West African Chimpanzees (*Pan troglodytes verus*) Inhabiting an Anthropogenic Landscape. Am. J. Primatol..

[109] Feedipedia Ear Maize. https://www.feedipedia.org/node/713.

[110] Bodnár K., Mousavi S.M.N., Nagy J. (2018). Evaluation of Dry Matter Accumulation of Maize *(Zea mays L*.) Hybrids. Acta Agrar. Debreceniensis.

[111] Wilson M.L., Hauser M.D., Wrangham R.W. (2007). Chimpanzees (*Pan troglodytes*) Modify Grouping and Vocal Behaviour in Response to Location-Specific Risk. Behaviour.

[112] Tarnaud L., Garcia C., Krief S., Simmen B. (2010). Apports nutritionnels, dépense et bilan énergétiques chez l’homme et les primates non-humains: Aspects méthodologiques1. Rev. Primatol..

[113] Pontzer H. (2015). Energy Expenditure in Humans and Other Primates: A New Synthesis. Annu. Rev. Anthropol..

[114] Kaiyala K.J., Ramsay D.S. (2011). Direct Animal Calorimetry, the Underused Gold Standard for Quantifying the Fire of Life. Comp. Biochem. Physiol. Part A Mol. Integr. Physiol..

[115] Hoogwerf B.J., Goetz C. (1983). Urinary C-Peptide: A Simple Measure of Integrated Insulin Production with Emphasis on the Effects of Body Size, Diet, and Corticosteroids*. J. Clin. Endocrinol. Metab..

[116] Sherry D.S., Ellison P.T. (2007). Potential Applications of Urinary C-Peptide of Insulin for Comparative Energetics Research. Am. J. Phys. Anthropol..

[117] Valé P.D., Béné J.-C.K., N’Guessan A.K., Crockford C., Deschner T., Koné I., Girard-Buttoz C., Wittig R.M. (2021). Energetic Management in Wild Chimpanzees (*Pan troglodytes verus*) in Taï National Park, Côte d’Ivoire. Behav. Ecol. Sociobiol..

[118] Higham J.P., Girard-Buttoz C., Engelhardt A., Heistermann M. (2011). Urinary C-Peptide of Insulin as a Non-Invasive Marker of Nutritional Status: Some Practicalities. PLoS ONE.

[119] Minetti A.E., Moia C., Roi G.S., Susta D., Ferretti G. (2002). Energy Cost of Walking and Running at Extreme Uphill and Downhill Slopes. J. Appl. Physiol..

[120] Campbell M.J., Dennison P.E., Butler B.W., Page W.G. (2019). Using Crowdsourced Fitness Tracker Data to Model the Relationship between Slope and Travel Rates. Appl. Geogr..

[121] Hill L., Swain D., Hill E. (2008). Energy Balance during Backpacking. Int. J. Sports Med..

[122] Looney D.P., Santee W.R., Hansen E.O., Bonventre P.J., Chalmers C.R., Potter A.W. (2019). Estimating Energy Expenditure during Level, Uphill, and Downhill Walking. Med. Sci. Sports Exerc..

[123] Richmond P.W., Potter A.W., Santee W.R. (2015). Terrain Factors for Predicting Walking and Load Carriage Energy Costs: Review and Refinement. J. Sport Hum. Perform..

[124] Schmitt C.A., Rich A.M., Parke S.-A.R., Blaszczyk M.B., Cramer J.D., Freimer N.B., Grobler J.P., Turner T.R. (2020). Anthropogenic Food Enhancement Alters the Timing of Maturational Landmarks among Wild Savanna Monkeys (*Chlorocebus pygerythrus*). bioRxiv.

[125] Leith D.A., Mpofu B.S., van Velden J.L., Reed C.C., van Boom K.M., Breed D., Kohn T.A. (2020). Are Cape Peninsula Baboons Raiding Their Way to Obesity and Type II Diabetes?—A Comparative Study. Comp. Biochem. Physiol. Part A Mol. Integr. Physiol..

[126] Krief S., Berny P., Gumisiriza F., Gross R., Demeneix B., Fini J.B., Chapman C.A., Chapman L.J., Seguya A., Wasswa J. (2017). Agricultural Expansion as Risk to Endangered Wildlife: Pesticide Exposure in Wild Chimpanzees and Baboons Displaying Facial Dysplasia. Sci. Total Environ..

[127] Spirhanzlova P., Fini J.-B., Demeneix B., Lardy-Fontan S., Vaslin-Reimann S., Lalere B., Guma N., Tindall A., Krief S. (2019). Composition and Endocrine Effects of Water Collected in the Kibale National Park in Uganda. Environ. Pollut..

[128] Wrangham R.W., Chapman C.A., Chapman L.J. (1994). Seed Dispersal by Forest Chimpanzees in Uganda. J. Trop. Ecol..

[129] Bakker J.P., Poschlod P., Strykstra R.J., Bekker R.M., Thompson K. (1996). Seed Banks and Seed Dispersal: Important Topics in Restoration Ecology. Acta Bot. Neerl..

[130] Ordaz-Németh I., Sop T., Amarasekaran B., Bachmann M., Boesch C., Brncic T., Caillaud D., Campbell G., Carvalho J., Chancellor R. (2021). Range-wide Indicators of African Great Ape Density Distribution. Am. J. Primatol..

