# Supplementary material for: All-You-Can-Eat: Influence of Proximity to Maize Gardens on the Wild Diet and the Forest Activities of the Sebitoli Chimpanzee Community in Kibale National Park
Source: Animals (Basel). 2022 Mar 22;12(7):806. doi: 10.3390/ani12070806 (PMC8996920; doi:10.3390/ani12070806)
Supplement: Supplementary file 1 [file animals-12-00806-s001.zip › animals-1568986-supplementary.pdf]

animals

MDPI

All-You-Can-Eat: Influence of Maize Gardens' Proximity on the Wild Diet and the Forest Activities of the Sebitoli Chimpanzee Community in Kibale National Park

Chloé Couturier*, Sarah Bortolamiol, Sylvia Ortmann, John-Paul Okimat, Edward Asalu and Sabrina Krief

* Correspondence: chloe.couturier@mnhn.fr

Supplementary Materials:

Table S1: List of chimpanzees monitored by the FNN protocol between January 2016 and January 2019 in Kibale National Park, Uganda.

Table S2: Repartition of FNN among sex-age classes, maize seasons and wild fruit availability between January 2016 and January 2019 in Kibale National Park, Uganda.

Table S3: Detailed statistics between sex-age classes in activity budget, daily paths, energy expenditures, frugivory, energy intakes and energy balance.

Text S1: Nutritional analysis protocol.

Table S4: List of wild fruits consumed by the Sebitoli community between January 2016 and January 2019 in Kibale National Park, Uganda.

Table S5: Dry mass (g) of 13 fruits from Sebitoli and Kanyawara in Kibale National Park, Uganda.

References:

54. N'guessan, A.K.; Ortmann, S.; Boesch, C. Daily Energy Balance and Protein Gain Among *Pan troglodytes verus* in the Taï National Park, Côte d'Ivoire. *Int. J. Primatol.* **2009**, *30*, 481–496. https://doi.org/10.1007/s10764-009-9354-1.

94. Houle, A.; Wrangham, R.W. Contest Competition for Fruit and Space among Wild Chimpanzees in Relation to the Vertical Stratification of Metabolizable Energy. *Animal Behaviour.* **2021**, *175*, 231–246. https://doi.org/10.1016/j.anbehav.2021.03.003.

103. Houle, A.; Conklin-Brittain, N.L.; Wrangham, R.W. Vertical Stratification of the Nutritional Value of Fruit: Macronutrients and Condensed Tannins: Intratree Variations in Macronutrients. *Am. J. Primatol.* **2014**, *76*, 1207–1232. https://doi.org/10.1002/ajp.22305.

131. Van Soest, P.J. *Nutritional Ecology of the Ruminant*; Cornell University Press: Ithaca, NY, USA, 2018.

Table S1. List of chimpanzees monitored by the FNN protocol between January 2016 and January 2019 in Kibale National Park, Uganda. .

Code ID	Sex	Estimated Date of Birth	Class[a]	Mother Status	Total Time of Observation	No. of focals[b]	No. of Focals (80%)[c]
ET	M	1983	AM		309:23	35	19
BM	M	1997	AM		251:07	32	17
AG	M	1997	AM		226:07	27	11
UL	M	2001	AM		156:15	19	7
KT	F	1975	MO	Gestant until 2016/8 then lactant	135:54	17	11
NE	M	1965	AM		120:33	16	12
LA	M	2000	AM		91:41	11	6
KE	F	1987	MO	Lactant until 2017/4 gestant until 2017/11 then lactant	81:07	10	6
KI	M	2005	YO		61:00	7	3
MU	M	1994	AM		44:44	5	3
KB	M	2005	YO		40:31	5	3
GB	F	1990	MO	Gestant until 2016/8 then lactant	37:41	5	2
DT	F	2000	AF		28:40	4	4
PP	F	1980	AF		27:39	4	1
MJ	F	1990	MO	Lactant until 2019	17:25	2	1
LK	M	2006	YO		16:29	2	2
HU	M	1993	AM		15:22	2	2
BR	F	1990	MO	Lactant until 2018	10:23	1	0
GL	F	1975	AF		8:01	1	1
NK	F	1985	MO	Lactant until 2018/5 then pregnant	6:42	1	1
Total					1686:44	206	112

[a] No individual changed during the study period; [b] FNN with a duration superior or equal to 6 hours; [c] Only FNN with at least 6 hours duration and 80% of the total feeding time covered by the 11 fruits used in nutritional analysis; AF: non-maternal adult females; MO: mothers; AM: adult males; YO: subadult males. Time of observation is expressed in hours and minutes.

Table S2. Repartition of FNN among sex-age classes, maize seasons and wild fruit availability between January 2016 and January 2019 in Kibale National Park, Uganda.

			**No maize**	**Maize**	**HFA**	**LFA**
All	Females	AF	6	3	2	7
		MO	17	19	14	20
	Males	AM	82	65	61	75
		YO	9	5	2	12
	Total		114	92	79	114
80%*	Females	AF	2	4	1	5
		MO	10	11	7	13
	Males	AM	37	40	34	38
		YO	4	4	2	6
	Total		53	59	44	62

* Only FNN with at least 80% of the total feeding time covered by the 11 fruits used in nutritional analysis; AF: non-mother adult females; MO: mothers; AM: adult males; YO: subadult males; HFA: high fruit availability; LFA: low fruit availability for wild fruits.

Table S3. Detailed statistics between sex-age classes in activity budget, daily paths, energy expenditures, frugivory, energy intakes and energy balance.

**Variables**		**Sex-Age classes**	
		**Kruskal–Wallis**	**Dunn and Bonferroni Cor.**
Activity Budget			
	% Rest	H(3) = 11.6<br>***p* <0.01***	**AM vs. MO: *p* <0.01***
	% Travel	H(3)= 15.3<br>***p* <0.005****	**AM vs. MO: *p* <0.005****<br>**YO vs. MO: *p* <0.05***
	% Feed	H(3) = 3.9<br>*p* = 0.272	-
DLT		H(3) = 25.4<br>***p* <0.0001*****	**MO vs. AM: *p* <0.01***
TDEE		H(3)= 79.2<br>***p* <0.0001*****	**MO vs. AM: *p* <0.0001*****<br>**MO vs. YO: *p* <0.0001*****<br>**MO vs. AF: *p* <0.0001*****<br>**AF vs. AM: *p* <0.0005*****<br>**AF vs. YO: *p* <0.05***
% Frugivory		H(3) = 0.22<br>*p* = 0.974	-
Ingestion rate		H(3) = 1.8<br>*p* = 0.614	-
Energy balance		H(3) = 1.28<br>*p* = 0.734	-

DLT: daily length travel, TDEE: total daily energy expenditures; AF: Non-suited adult females; AM: adult male; MO: mothers; YO: subadult males. Bold *p*-values are significant (* <0.05 ** <0.005, *** <0.0005).

Text S1. Nutritional analysis protocol

Diet quality was analyzed as previously described in N'Guessan et al. [54]. Briefly, we determined in duplicate: gross energy (kJ/g); dry matter DM (%); macronutrients (%DM) and minerals (mg/kgDM).

For nutrient content analyses, we used the following standard methods: Dry matter was determined by drying a subsample at 105 °C until constant weight. Gross energy was determined via bomb calorimetry (IKA-Calorimeter C4000, Ika, Stauffen, Germany) and crude fat via ethyl ether extraction (Soxhlett method; AOAC no. 963.15). For crude protein analysis (CP, AOAC no. 977.02), a subsample was burned at high temperature (about 950 °C) in pure oxygen (Dumas combustion). This provides the nitrogen content of the sample, and crude protein was calculated according to CP = N x 6.25. Starch, sucrose, glucose, and fructose were determined with the help of enzymatic tests and crude ash by burning the dried sample in a muffle furnace for at least two hours at 550 °C (AOAC no. 942.05), after which only inorganic matter remained; the amount of ash was then obtained by the difference in weights. The detergent system of fiber analysis after van Soest [131] was used to estimate the amount of indigestible carbohydrates in samples. This method consists of dissolving successively soluble cell components, hemicelluloses, and celluloses. We obtained sequentially neutral detergent fiber (NDF, AOAC no. 2002.04), acid detergent fiber (ADF), and acid detergent lignin (ADL, AOAC no. 973.18). For mineral analysis (Ca; Cu; Fe; Zn; Mg; Mn; Na, K, P, S, and Zn), samples were microwave digested and analyzed by inductively coupled plasma optical emission spectrometer (model Optima 8000, Perkin Elmer).

Table S4. List of wild fruits consumed by the Sebitoli community between January 2016 and January 2019 in Kibale National Park, Uganda.

Species	Part Eaten	Feeding Time (h)	% (fruits)	% (total diet)
Ficus sur (F. capensis)	**URF, RF**	**60.46**	**18.3**	**15.0**
Ficus dawei (F. saussureana)	**RF**	**59.80**	**18.1**	**14.8**
Ficus exasperata	**URF, RF**	**43.08**	**13.0**	**10.7**
Ficus natalensis	**URF, RF**	**34.63**	**10.5**	**8.6**
Cordia abyssinica (C. africana)	**RF**	**29.19**	**8.8**	**7.2**
Mimusops bagshawei (M. ugandensis)	**URF, RF**	**22.20**	**6.7**	**5.5**
Drypetes sp. (D. gerradii or *D.battiscombei)**	RF	20.76	6.3	5.1
Ficus brachylepis (F. sansibarica)	**RF**	**13.72**	**4.1**	**3.4**
Aphania senegalensis (Lepisanthus senegalensis)	**RF**	**9.74**	**2.9**	**2.4**
Ficus mucoso	**RF**	**8.72**	**2.6**	**2.2**
Ficus cyathistipula(F. callenscens)	RF	5.15	1.6	1.3
Cordia ugandensis(C. millenii)	RF	4.66	1.4	1.2
Phytolacca sp.	RF	3.64	1.1	0.9
Bosqueia phoberos (Trilepisium madagascariense)	RF	3.15	1.0	0.8
Aframomum sp.	URF, RF	2.59	0.8	0.6
Triclisia sp.	URF, RF	1.49	0.5	0.4
Carapa sp.	RF	1.01	0.3	0.3
Ficus thoningii	RF	1.01	0.3	0.3
Euadenia eminens	RF	0.71	0.2	0.2
Ficus vallis	RF	0.67	0.2	0.2
Urera hypsiloides	RF	0.66	0.2	0.2
Conopharyngia sp.	URF, RF	0.65	0.2	0.2
Tylostemon ugandensis	RF	0.52	0.2	0.1
Unspecified	RF	0.50	0.2	0.1
Dovyalis macrocalyx	RF	0.48	0.1	0.1
Pseudospondias microcarpa	RF	0.41	0.1	0.1
Pancovia turbinata	RF	0.38	0.1	<0.1
Ehretia cymosa	RF	0.19	<0.1	<0.1
Monodora sp.	RF	0.18	<0.1	<0.1
Ficus stipulifera	RF	0.12	<0.1	<0.1
Parinari holstii	RF	0.10	<0.1	<0.1
Hoslundia opposita	RF	0.01	<0.1	<0.1
Ficus urceolaris	RF	0.01	<0.1	<0.1

* Subspecies of Drypetes sp. among the two present in the study area were not specified by the observers. Otherwise, synonym species are specified in brackets. Species in bold were collected in 2015 for nutritional analysis and integrated with energy calculations for this study. URF: unripe fruit, RF: ripe fruit.

Table S5. Dry mass (g) of 13 fruits from Sebitoli and Kanyawara in Kibale National Park, Uganda.

Species	Part	SBL[1]		KWRA.3[2]	KWRA.4[3]
		N	X (SD)		
Aphania senegalensis	RF	250	0.89 (0.58)	-	-
Cordia abyssinica	RF	200	0.28 (0.24)	0.22	0.38
Ficus sansibarica	RF	40	7.14 (18.16)	2.66	2.66
Ficus sausureana	RF	336	1.70 (3.53)	-	-
Ficus exasperata	RF	100	0.22 (0.67)	-	0.26
	URF	50	0.42 (0.35)	-	0.17
Ficus mucoso	RF	71	5.88 (0.65)	-	-
Ficus natalensis	RF	350	0.41 (0.68)	0.31	0.32
	URF	-	-	-	-
Ficus sur	RF	1965	0.95 (3.79)	0.72	0.72
	URF	300	0.71 (1.75)	0.55	0.55
Mimusops bagshawei	RF	-	-	1.18	1.19
	URF	100	0.72 (0.65)	0.97	0.80

N: number of fruits, X: mean dry weight per fruit (g), SD: standard deviation. Values for the different sites are from: [1]this study; [2][94]; [3][103]; SBL: Sebitoli, KWRA: Kanyawara.
